# Advancements in the Treatment of Multiple Myeloma

**DOI:** 10.7759/cureus.74970

**Published:** 2024-12-02

**Authors:** Esteban Zavaleta-Monestel, Ricardo Quesada-Villaseñor, Monserrat Barrantes-López, Sebastián Arguedas-Chacón, Jeimy Campos-Hernández, Carolina Rojas-Chinchilla, Jonathan García-Montero, Josué Castro-Ulloa, Adriana Anchía-Alfaro, José Ricardo Montenegro-Chaves

**Affiliations:** 1 Pharmacy, Hospital Clinica Biblica, San Jose, CRI; 2 General Medicine, Hospital Clinica Biblica, San José, CRI; 3 Pharmacy, Universidad de Iberoamerica, San José, CRI; 4 Pharmacy, Hospital Clinica Biblica, San José, CRI; 5 Pharmacy, Universidad de Iberoamérica, San José, CRI; 6 Pharmacy, Hospital Clínica Bíblica, San José, CRI; 7 General Medicine, Hospital Clínica Bíblica, San José, CRI; 8 Hematology, Hospital Clínica Bíblica, San José, CRI

**Keywords:** bispecific monoclonal antibodies, chemotherapy, immunologic factors, multiple myeloma, neoplasms, proteasome inhibitors, therapeutics

## Abstract

Multiple myeloma (MM) is the second most common hematological malignancy globally. Despite its reputation as an aggressive and fatal disease, recent advancements in treatment have significantly improved patient outcomes. Over the past decade, innovative therapies with fewer side effects have emerged, including proteasome inhibitors (e.g., bortezomib, carfilzomib, ixazomib), monoclonal antibodies (e.g., daratumumab, isatuximab, elotuzumab), immunomodulators (e.g., thalidomide, lenalidomide, pomalidomide), and corticosteroids (e.g., dexamethasone). Additionally, combination regimens utilizing these agents have demonstrated improved efficacy by targeting multiple pathways simultaneously. These therapies are more precise in their ability to attack myeloma cells, leading to enhanced patient quality of life, prolonged remission, and delayed disease progression. This article explores the evolution of MM therapies, highlighting the potential for a cure through ongoing breakthroughs in drug development and combination treatments. The analysis underscores the optimism surrounding the future of MM treatment and the need for continued research to make MM a manageable and potentially curable disease.

## Introduction and background

Cancer affects populations worldwide with multiple myeloma (MM) standing out as a significant type. It is classified as a hematological cancer since it is characterized by the presence of a neoplasm of plasma cells that are established in the bone marrow [1]. MM has affected populations for centuries. In the past, its diagnosis was unclear due to limited knowledge and advances in cancer research. The disease was first described in the 19th century, with milestones such as the identification of Bence Jones proteins and the use of the term multiple myeloma to describe its hallmark bone lesions. The first case of MM in the United States was published in 1894, and since then, a great number of studies have started [2].

Multiple myeloma, also known as Kahler's disease, is a hematological malignancy originating from plasma cells in the bone marrow. These abnormal plasma cells multiply uncontrollably, leading to a wide range of health complications. It is the second most common hematological neoplasm according to reported cases. MM is defined by the presence of monoclonal plasma cells producing a paraprotein that can cause clinical complications, including anemia, renal failure, hypercalcemia, and bone lesions. Despite being an incurable disease, its outlook has greatly improved due to the advent of innovative treatments like thalidomide, lenalidomide, and bortezomib. Additionally, progress in supportive therapies, including dialysis, bisphosphonates, and advanced surgical procedures, along with multidisciplinary care, has led to longer survival times, frequently surpassing a median of five years [3].

Despite the availability of various diagnostic tests, the definitive diagnosis of MM is established based on a combination of clinical, laboratory, and pathological criteria. These include the presence of more than 10% clonal plasma cells in a bone marrow aspirate or a biopsy-proven plasmacytoma, along with evidence of end-organ damage related to MM (CRAB criteria: hypercalcemia, renal insufficiency, anemia, or bone lesions). Flow cytometry and advanced imaging techniques such as MRI or PET/CT further aid in confirming the diagnosis and assessing disease extent [4].

Patients with MM frequently experience symptoms such as severe bone pain, fatigue, and weight loss, often associated with bone marrow infiltration by clonal plasma cells and the presence of monoclonal protein in serum or urine. Additionally, bone lesions and immunodeficiencies are prevalent, contributing to significant morbidity. The diagnosis is typically established when there is evidence of organ damage directly linked to the plasma cell disorder, distinguishing it from precursor stages like monoclonal gammopathy of undetermined significance or smoldering multiple myeloma, which do not require immediate treatment [5].

In the absence of a definitive cure, MM carries a significant mortality rate if a strategy is not set. According to global statistics from the International Agency for Research on Cancer (IARC) and the World Health Organization (WHO), in 2022, there were 187,952 new diagnoses and 121,388 deaths associated with MM. In 2023, a total of 1,970 deaths were attributed to this disease [6].

Statistical data from the United States in 2023 reveals a higher prevalence of MM among men, both in terms of incidence and mortality. Out of the 35,730 new cases reported that year, over 50% occurred in men, and approximately 2,000 more men succumbed to the disease compared to women [7].

In various countries, the standard treatment for MM typically begins with triple or quadruple combination regimens. However, if the disease progresses, it is crucial to establish a follow-up strategy that includes effective second-line combinations, incorporating new therapeutic drugs to optimize patient outcomes [8].

Genetic investigations have revealed that neoplastic cells in MM frequently harbor mutations in the IgH and IgL chains. These mutations arise from somatic hypermutation processes associated with the disease. Furthermore, MM is characterized by translocations involving these two immunoglobulin types [9].

Despite the currently incurable nature of MM, numerous treatment options have emerged over the years, significantly improving patient life expectancy. Among the most employed therapies against myeloma cells are immunomodulatory agents, proteasome inhibitors, and monoclonal antibodies, all of which have contributed to extended survival, among other benefits for patients. Bone marrow transplants, often referred to as stem cell transplants, are another key therapeutic option, enabling high-dose chemotherapy to be administered by restoring healthy blood-forming cells [7].

This article aimed to analyze the evolution of therapeutic strategies for multiple myeloma, focusing on the transition from traditional treatments to novel targeted therapies. By consolidating advancements in drug development, genetic insights, and supportive care, the study highlights how these innovations have shaped patient management and outcomes, with the goal of optimizing treatment protocols and paving the way toward a potential cure.

## Review

Methodology

This review article was prepared through an exhaustive search of published scientific literature on the treatment of multiple myeloma. Databases such as PubMed, Scopus, and Google Scholar were consulted to identify relevant studies, systematic reviews, and clinical trials published in the last 10 years. The selection of articles was based on their relevance to the topic, methodological rigor, and impact on the development of current and future treatments for multiple myeloma. No statistical analysis or formal systematic review was conducted due to the descriptive nature of this narrative review article, which aimed to provide a comprehensive overview of therapeutic advances in the treatment of this disease.

**Figure 1:**
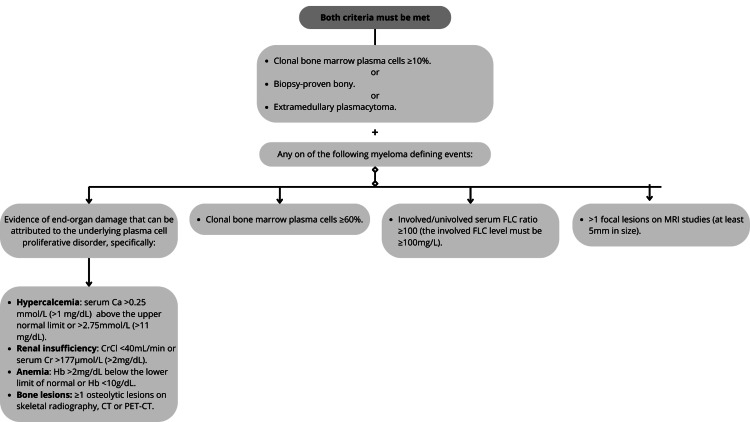
Criteria for multiple myeloma. Ca: calcium; CrCl: creatinine clearance; Hb: hemoglobin; CT: computed tomography; PET-CT: positron emission tomography; FCL: free light chain; MRI: magnetic resonance imaging The image is created by the authors of this study based on the information from Rajkumar [4].

MM patients typically progress through various stages as the disease unfolds and criteria change. The following graphic outlines these associated characteristics: as previously discussed, the available drugs for treating MM belong to distinct drug families, each with unique side effects, dosages, and mechanisms of action (Figure 1) [10]. These treatments, due to their diverse mechanisms of action, can be combined to create a more comprehensive treatment approach with higher success rates.

As the disease progresses, bone involvement becomes increasingly severe, ranging from generalized osteoporosis to localized bone lesions with fractures. In advanced stages, MM can lead to complications such as kidney damage primarily caused by the deposition of monoclonal light chains (Bence Jones proteins) in renal tubules, leading to cast nephropathy. Other mechanisms include hypercalcemia-induced nephrocalcinosis, amyloid deposition, and chronic interstitial nephritis. These renal complications often coexist with more severe bone disease [11].

The diagnostic process for MM is multifaceted, utilizing blood and urine analyses to quantify M proteins, which are abnormal immunoglobulins secreted by myeloma cells. Bone marrow biopsy remains essential to confirm clonal plasma cell proliferation, while imaging studies, such as PET/CT and MRI, provide insights into bone involvement and disease extent. These tools not only establish the diagnosis but also guide treatment planning by identifying disease stages and complications [12,13].

**Figure 2:**
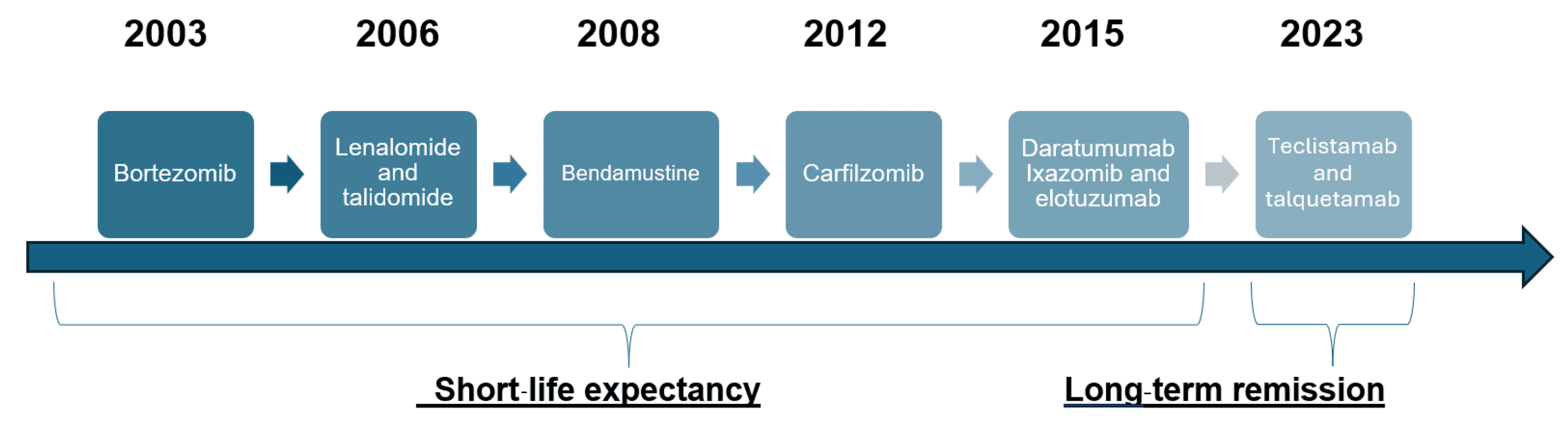
Chronology of approval for major MM treatments by FDA. Chronology of FDA-approved treatments for multiple myeloma. This timeline illustrates the FDA approval dates for major multiple myeloma treatments as follows: bortezomib (2003), lenalidomide and thalidomide (2006), bendamustine (2008), carfilzomib (2012), daratumumab, ixazomib, and elotuzumab (2015), teclistamab, and talquetamab (2023). Treatments approved between 2003 and 2015 are indicated as having limited life expectancy (“short life expectancy”), while those from 2023 are associated with a potential for long-term remission (“long-term remission”). The image is created by the authors of this study. MM: multiple myeloma

Despite the incurable nature of MM, ongoing research and development have led to the introduction of novel therapies and the refinement of existing treatments, significantly improving the quality of life and extending the survival of patients. The following figure summarizes the key treatment options for MM that have emerged over the years (Figure 2) [14-17].

The treatment landscape for multiple myeloma (MM) has expanded significantly, with various therapeutic options targeting different pathways and mechanisms. Among these, lenalidomide (Revlimid), a less toxic derivative of thalidomide, has emerged as a key agent. Initially approved by the FDA in 2006 for use with dexamethasone in patients with relapsed or refractory MM, lenalidomide's role has since evolved. It is now a standard maintenance therapy following autologous stem cell transplantation (ASCT). These studies demonstrated a significant improvement in progression-free survival (PFS), extending it by nearly 20 months compared to placebo. This maintenance approach has become integral to prolonging remission and enhancing overall survival, albeit with careful consideration of associated risks, such as secondary malignancies [14,16,17].

In addition, the approval of carfilzomib (Kyprolis) in combination with lenalidomide and dexamethasone has further exemplified the benefits of multidrug regimens. Clinical trials have shown that this three-drug combination achieves deeper, more durable responses compared to dual-drug therapy, increasing PFS from 17.6 to 23.6 months. Such findings underline the importance of synergistic strategies in addressing the challenges of relapse, a near-universal event in MM [14,16,17].

Other agents, such as bendamustine, also play a vital role in the treatment paradigm. This bifunctional alkylating agent, which acts as a purine analog, has demonstrated efficacy in various hematological malignancies, including non-Hodgkin lymphomas and chronic lymphocytic leukemia. Bendamustine's unique mechanism of action involves inducing DNA damage and mitotic catastrophe, making it a valuable option for patients with resistant or recurrent diseases. The growing arsenal of therapies underscores the importance of personalized, combination-based approaches to optimize outcomes in multiple myeloma [14,16,17]. The following table provides a comparative overview of the different pharmacological groups and their primary medications for MM, along with their respective mechanisms of action (Table 1) [15,18-30].

**Table 1:** Types of treatments and their characteristics for MM. MM: multiple myeloma

Pharmacological group	Mechanism of action and characteristics
Chemotherapy	Bendamustine: this cross-links DNA strands, affecting their synthesis and repair. Acts on cells in both resting and dividing phases. Typically administered in two treatment cycles for MM.
Corticosteroids	Dexamethasone: this inhibits phospholipase A2, thereby affecting prostaglandin synthesis with anti-inflammatory effects. Often used as complementary medication in treating the disease.
Immunomodulators	Thalidomide: this has various functions, including angiogenesis inhibition and apoptosis of cancer cells, though its specific mechanism of action is not fully defined. Administered orally daily in 12-cycle treatments. Lenalidomide: this inhibits angiogenesis, modulates the immune system, and induces apoptosis of cancer cells. Administered orally in 28-day cycles.
Proteasome inhibitors	Bortezomib: this irreversibly inhibits the proteasome, leading to apoptosis of myeloma cells by inhibiting nuclear factor kappa B. Carfilzomib: this irreversibly binds to the β5 and β7 subunits of the proteasome.
Monoclonal antibodies	Denosumab: this binds to RANKL, inhibiting receptor activation, and osteoclast formation and function. Daratumumab: this binds to CD38 protein, delaying or halting the growth of cancer cells.

**Figure 3:**
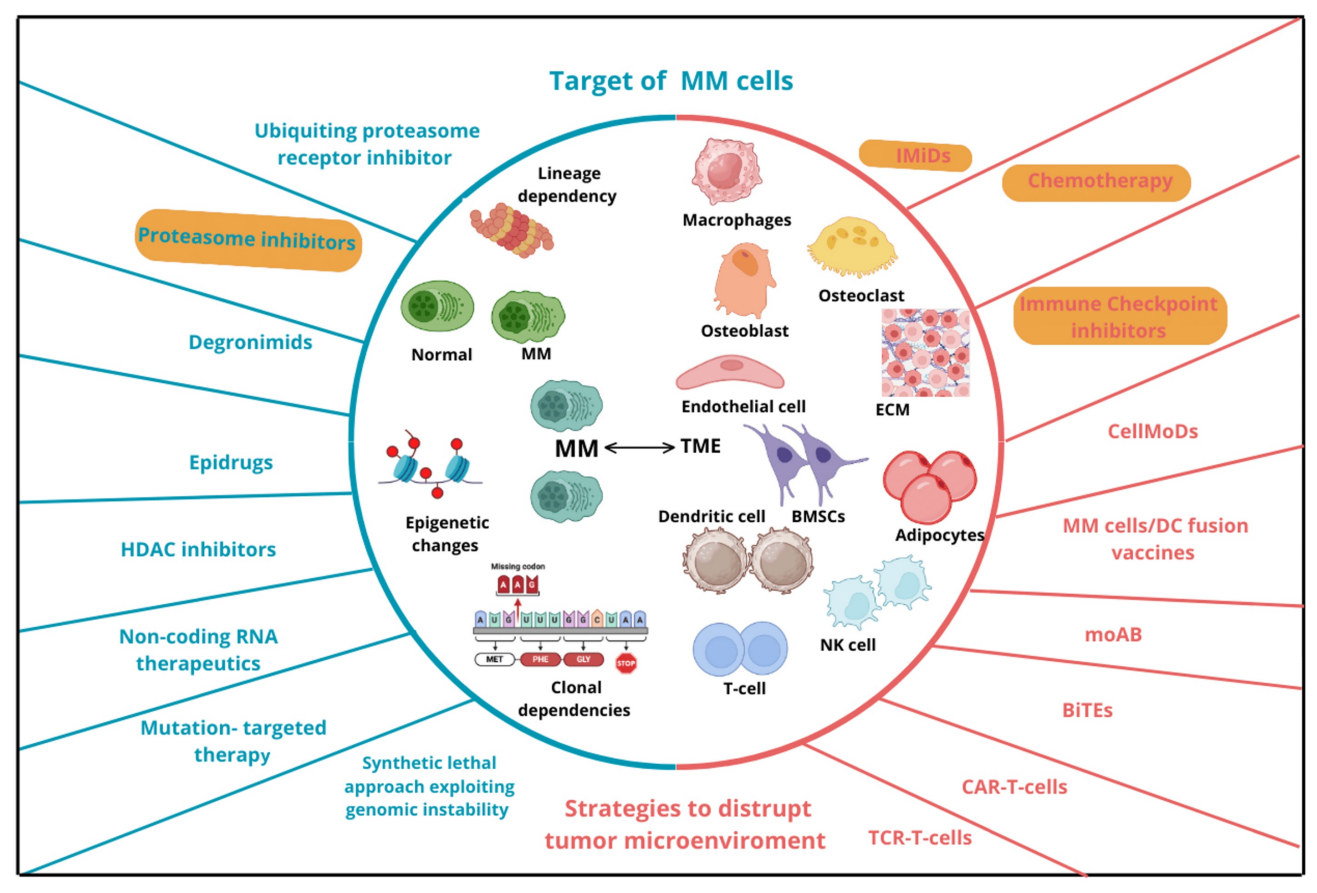
Treatments for MM according to therapeutic approaches. Ca: calcium; CrCl: creatinine clearance; Hb: hemoglobin; CT: computed tomography; PET-CT: positron emission tomography; FCL: free light chain; MRI: magnetic resonance imaging; MM: multiple myeloma The image is created by the authors of this study based on information from Solimando et al. (CCBY 4.0) [31].

Figure 3 summarizes the approach that recent treatments have. On the left side of the picture, treatments such as proteasome inhibitors (e.g., bortezomib) and others are shown, demonstrating those therapies targeting the toxicity of MM cells. Treatments on the right side of the picture enlist those that focus on altering the dynamics of the MM cell with the tumor microenvironment. Among these, chemotherapies, immunomodulators such as thalidomide, and immune checkpoint inhibitors, stand out [31].

**Figure 4:**
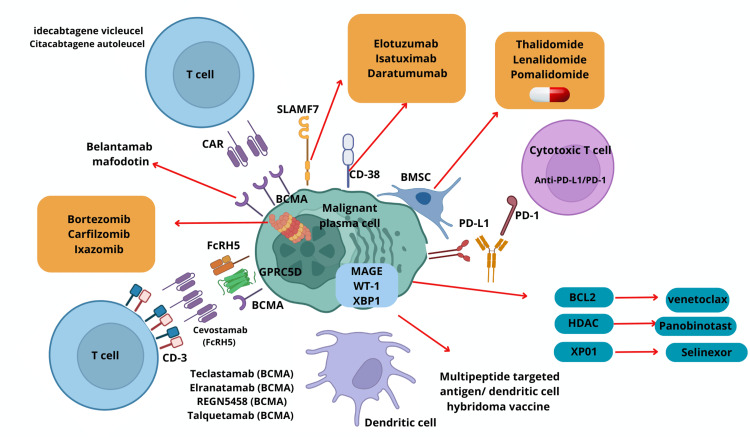
Mechanism of action of therapies for MM. CAR: chimeric antigen receptor; SLAMF7: surface antigen CD319; BCMA: B cell maturation antigen; BMSC: bone marrow stromal cell; PD-L1: programmed death-ligand 1; GPRC5D: G protein-coupled receptor family C group 5 member D; FcRH5: Fc receptor homolog 5; MAGE: melanoma-associated antigen; WT-1: Wilms tumor 1; XBP1: X-box binding protein 1; BCL2: B cell lymphoma-2; HDAC: histone deacetylases; XP01: exportin-1; MM: multiple myeloma The image is created by the authors of this study based on information from Solimando et al. (CCBY 4.0) [31].

Figure 4 illustrates the mechanisms of action of the treatments explained in Table 1 in a more visual fashion. The interaction between the MM cell, the T cells, other cells, and protein targets is illustrated, as well as the targets of the drugs studied.

In the case of bendamustine as a chemotherapy treatment, it used to be the first choice for MM cases. However, over the years, innovation has led to other medications that are more specific at attacking myeloma cells, reducing collateral damage compared to traditional chemotherapy. Additionally, monoclonal antibodies bind to the CD38 protein. They are generally administered intravenously in combination with other medications, effectively eliminating cancer cells directly and enhancing immune system attacks on them [29].

Drugs belonging to proteasome inhibitors include bortezomib, which was the first approved drug used in MM treatment. Subsequent generations of these drugs have shown improvement in reducing both the quantity and severity of adverse effects observed in patients. It is also noteworthy that carfilzomib is currently under study for possible triple combination therapies in the early stages of the disease [30].

Other treatments available are immunomodulators such as thalidomide, a medication that had a tragic background and significant side effects on a significant number of patients. However, its use as a treatment for MM has been widely studied, and now it represents a safe option for patients with this disease. This medication is dose-dependent, so close monitoring of the patient response is necessary to make any dose adjustments and avoid adverse effects [32].

All these drugs have been studied and have shown that combining them in treatment has been much more effective than using them as monotherapy. Likewise, it has been observed that their combination is able to reduce the occurrence of relapses and improve life expectancy. Below is a table demonstrating the different medications commonly used combined as a treatment for this condition (Table 2) [29,33,34].

**Table 2:** Treatments for MM used in combination. MM: multiple myeloma

Medications commonly used in combination to treat MM
1. Lenalidomide (or thalidomide) and dexamethasone
2. Carfilzomib (or bortezomib), lenalidomide, and dexamethasone
3. Bortezomib (or carfilzomib), cyclophosphamide, and dexamethasone
4. Elotuzumab (or daratumumab), lenalidomide, and dexamethasone
5. Elotuzumab, bortezomib, and dexamethasone
6. Belantamab mafodotin, bortezomib, and dexamethasone

It is also important to highlight that MM treatments are used in different phases of the disease, such as frontline and maintenance therapy. Initially, the treatment would typically involve immunomodulatory drugs or bortezomib either as monotherapy or in combination, aiming to control the disease and reduce the risk of early mortality. Once treatment transitions to maintenance therapy, the goal is for patients to remain in remission for as long as possible with the best achievable quality of life. During this phase, chemotherapies, a proteasome inhibitor, or immunomodulators combined with corticosteroids are often used [35,36].

Each of these drugs, either as monotherapy or combined, has been tested in patients, showing good results in improving quality of life and controlling the disease. Below is a table demonstrating various studies or clinical cases that prove this effectiveness (Table 3) [37-41].

**Table 3:** Clinical studies demonstrating the efficacy of treatments for MM. MM: multiple myeloma

Studies	Title	Outcomes
Moreau et al., 2016 [37]	Oral Ixazomib, lenalidomide, and dexamethasone for multiple myeloma	A total of 722 patients were randomly treated with Ixazomib + lenalidomide + dexamethasone or with placebo. The treatment demonstrated greater expectation of improvement in patients over a period of 20 months
Stewart et al., 2015 [38].	Carfilzomib, lenalidomide, and dexamethasone for relapsed multiple myeloma	It was proven that the use of lenalidomide + dexamethasone + carfilzomib achieved a higher range of survival when used together instead of carfilzomib alone
Palumbo et al., 2016 [39]	Daratumumab, bortezomib, and dexamethasone for multiple myeloma	The combination of daratumumab + bortezomib and dexamethasone had a greater positive effect on patients after one year of follow-up treatments
Mateos et al., 2019 [40].	Pembrolizumab plus pomalidomide and dexamethasone for patients with relapsed or refractory multiple myeloma (KEYNOTE- 183): a randomised, open-label, phase 3 trial	A total of 249 patients were randomly assigned, with one group receiving pembrolizumab alone and another group receiving a combination of pembrolizumab, dexamethasone, and pomalidomide, with the latter showing better results
Kubo et al., 2020 [41]	Elotuzumab plus lenalidomide and dexamethasone for newly diagnosed multiple myeloma: a randomized, open-label, phase 2 study in Japan	The efficacy and safety of elotuzumab were evaluated, marking the first attempt to verify the use of eight cycles of the medication, resulting in a positive outcome

For a long time, research has been conducted and different drugs have been tested to find a cure for MM. However, despite significant advancements and the appearance of new therapies, it seems unavoidable for patients to continue experiencing relapses, and the disease remains fatal. A cure has still not been found [42-44].

It is important to note that even with all these available medications, MM remains a disease with high mortality rates because, as mentioned, there is no treatment that has successfully cured the disease entirely. According to studies, in the United States, Australia, and Europe, mortality rates have been increasing. In 2021, a mortality rate of 94% was recorded. Thus, not only has the incidence of the disease increased over the years, but mortality rates have also risen [45].

## Conclusions

The treatment landscape for multiple myeloma (MM) has undergone remarkable advancements, significantly improving patient survival and quality of life. Targeted therapies and combination regimens, such as lenalidomide, carfilzomib, and bendamustine, have transformed disease management, providing deeper and more durable responses while minimizing adverse effects. These therapies have extended progression-free survival and are increasingly integral to maintenance strategies post-transplantation.

While a definitive cure for MM remains elusive, the progress made thus far underscores the potential to transform MM into a manageable disease. Ongoing innovation in treatment combinations and robust clinical trials exploring synergistic effects are vital to achieving this goal, offering patients not only longer lives but also better quality of life.
